# Piloting an ICU follow-up clinic to improve health-related quality of life in ICU survivors after a prolonged intensive care stay (PINA): feasibility of a pragmatic randomised controlled trial

**DOI:** 10.1186/s12871-023-02255-1

**Published:** 2023-10-14

**Authors:** Karl Philipp Drewitz, Claudia Hasenpusch, Christine Bernardi, Susanne Brandstetter, Christoph Fisser, Katharina Pielmeier, Magdalena Rohr, Vreni Brunnthaler, Konrad Schmidt, Maximilian V. Malfertheiner, Christian J. Apfelbacher

**Affiliations:** 1 Institute of Social Medicine and Health Systems Research, Medical Faculty, Otto von Guericke University Magdeburg, Leipziger Str. 44, 39120 Magdeburg, Germany; 2 Medical Sociology, Institute of Epidemiology and Preventive Medicine, University of Regensburg, Dr.-Gessler-Str. 17, 93051 Regensburg, Germany; 3 University Children’s Hospital Regensburg, University of Regensburg, Klinik St. Hedwig, Steinmetzstr. 1-3, 93049 Regensburg, Germany; 4 Department of Internal Medicine II, University Hospital Regensburg, Franz-Josef-Strauss-Allee 11, 93053 Regensburg, Germany; 5 Caritas-Krankenhaus St. Josef, Landshuter Str. 65, 93053 Regensburg, Germany; 6 Institute of General Practice and Family Medicine, Charité – Universitätsmedizin Berlin, Charitéplatz 1, 10098 Berlin, Germany; 7 Institute of General Practice and Family Medicine, Jena University Hospital, Bachstr. 18, 07743 Jena, Germany; 8 Klinik Donaustauf, Ludwigstr. 68, 93093 Donaustauf, Germany; 9 Family Medicine and Primary Care, Lee Kong Chian School of Medicine, Nanyang Technological University, 50 Nanyang Avenue, Singapore, 639798 Singapore

**Keywords:** Complex intervention, Critical care, ICU follow-up clinic, Mechanical ventilation, Pilot study, Post-intensive care syndrome (PICS)

## Abstract

**Background:**

ICU survivors often suffer from prolonged physical and mental impairments resulting in the so called “Post-Intensive Care Syndrome” (PICS). The aftercare of former ICU patients affected by PICS in particular has not been addressed sufficiently in Germany so far. The aim of this study was to evaluate the feasibility of a pragmatic randomised trial (RCT) comparing an intensive care unit (ICU) follow-up clinic intervention to usual care.

**Methods:**

This pilot study in a German university hospital evaluated the feasibility of a pragmatic RCT. Patients were assigned in a 1:1 ratio to an ICU follow-up clinic intervention or to usual care. The concept of this follow-up clinic was previously developed in a participatory process with patients, next of kin, health care professionals and researchers. We performed a process evaluation and determined acceptability, fidelity, completeness of measurement instruments and practicality as feasibility outcomes. The RCT’s primary outcome (health-related quality of life) was assessed six months after ICU discharge by means of the physical component scale of the Short-Form-12 self-report questionnaire.

**Results:**

The pilot study was conducted from June 2020 to May 2021 with 21 and 20 participants in the intervention and control group. Principal findings related to feasibility were 85% consent rate (*N* = 48), 62% fidelity rate, 34% attrition rate (*N* = 41) and 77% completeness of outcome measurements. The primary effectiveness outcome (health-related quality of life) could be measured in 93% of participants who completed the study (*N* = 27). The majority of participants (85%) needed assistance with follow-up questionnaires (practicality). Median length of ICU stay was 13 days and 85% (*N* = 41) received mechanical ventilation, median Sequential Organ Failure Assessment Score was nine. Six-month follow-up assessment was planned for all study participants and performed for 66% (*N* = 41) of the participants after 197 days (median).

**Conclusion:**

The participatory developed intervention of an ICU follow-up clinic and the pragmatic pilot RCT both seem to be feasible. We recommend to start a pragmatic RCT on the effectiveness of the ICU follow-up clinic.

**Trial registration:**

ClinicalTrials.gov US NLM, NCT04186468, Submission: 02/12/2019, Registration: 04/12/2019, https://clinicaltrials.gov/ct2/show/NCT04186468

**Supplementary Information:**

The online version contains supplementary material available at 10.1186/s12871-023-02255-1.

## Background

Survivors of ICU (intensive care unit) treatment often suffer from a wide range of physical, cognitive and mental health difficulties, referred to as post-intensive care syndrome (PICS) [1–3]. Available studies and data differ with respect to the prevalence and characteristics of PICS in ICU survivors [4–7]. For example, 25–80% of ICU survivors experience intensive care unit-acquired weakness (ICUAW) [8, 9], 30–80% cognitive impairments [4, 10, 11] and about 50% depression, anxiety or posttraumatic stress disorder (PTSD) [4, 6, 7]. Physical, cognitive and mental impairments have negative effects on patients’ health-related quality of life (HRQOL) [7, 12, 13]. A lower HRQOL compared to the general population is still present one year after discharge from ICU [12, 14, 15]. In addition, several studies show evidence for complications including depressive, anxiety and posttraumatic stress disorder also in family members, which is conceptualized as post-intensive care syndrome family (PICS-F) [9].

**Fig. 1:**
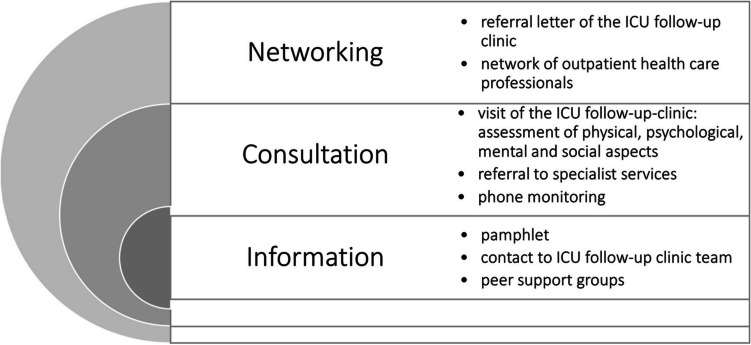
Main components of the ICU follow-up clinic adapted from our study protocol [28]

To address adverse sequelae of former critically ill patients as well as of their next of kin, several aftercare programs such as ICU follow-up clinics were developed. A recent systematic review on effects of ICU follow-up clinics concluded that symptoms of depression and mental HRQOL may be improved by this kind of intervention [16] but the quality of evidence regarding the effectiveness of those clinics is low [17–19]. Existing concepts of ICU follow-up clinics differ in the management (e.g. led by nurses, physicians or a multidisciplinary team), the focus of the intervention (e.g. physical and/or mental), the administration of consultation and counselling (direct contact or by phone), the frequency/dose of follow-up (e.g. weekly or monthly) or the eligible patient group (e.g. duration of ICU stay or specific diagnosis). This makes comparisons regarding effectiveness of interventions challenging [17–22]. While some European countries already have integrated ICU follow-up clinics in several hospitals [18, 20], no follow-up services are available for critically ill patients after discharge from ICU in Germany until now and the internationally implemented concepts have not been developed with intense involvement of all relevant stakeholder groups. Rehabilitation in Germany is rather specialized to disciplines and specific organ failures, than to general ICU population [23]. In general, ICU patients with severe underlying diseases are transferred to specialized rehabilitation facilities for early rehabilitation according to the level of support they require. In contrast to early rehabilitation, follow-up rehabilitation is part of medical rehabilitation that takes place when there is no (longer) need for acute medical treatment. The patients are already early mobilised and capable of self-help. The follow-up rehabilitation can be either conducted ambulatory, in a specialized rehabilitation centre or home-based [24]. However, after rehabilitation the support for further treatment is left to the treating physician without special experience for those patients with severe underlying diseases. Therefore, specialized follow-up services for those patients are necessary to provide the best-possible care [25]. Against this background, a multidisciplinary stakeholder group composed of health care professionals, researchers, patients and next of kin conceptualized an ICU follow-up clinic intervention in a German university hospital. The intervention was developed in a participatory process as previously reported [26–28] and consists of three main components: information, consultation and networking (Fig. 1). In brief, the development included face-to-face interviews with former ICU patients, next of kin of former ICU patients, six focus group discussions and six expert interviews to capture all relevant stakeholders’ perspectives (in total, nine different professions). Results of interviews, discussions, evidence from the literature and evidence from claims data analysis were assessed and synthesised to create a first draft of the intervention. This draft was discussed and refined in two workshops with stakeholders and finalised by the interdisciplinary project team.

## Methods/Study design

### Trial design

The methodology of the study has been outlined previously [28]. Briefly, our study was a pragmatic, single-centre, superiority, two-armed pilot randomised controlled trial with 1:1 allocation ratio, conducted between December 2019 and June 2021. To explore feasibility and acceptability of the trial and the intervention, it was accompanied it by a mixed-methods process evaluation according to the Medical Research Council (MRC) framework [29, 30] performed by a scientific study team not involved in the clinical study. Quantitative as well as qualitative data were collected and analysed alongside the pilot RCT. The institutional Ethics Committee of the University of Regensburg approved the study on 26/09/2019 (19–1522-101). Every participant actively agreed to be part of the study and informed consent was obtained according to the Helsinki Declaration [31]. We registered the study at a trials registry (clinicaltials.gov, NCT04186468) on 04/12/2019 before inclusion of the first participant.

### Participants and randomisation

The clinical study team (two physicians and two nurses) screened eligible patients on a daily basis at three ICUs (two medical and one surgical ICU) of Regensburg University Hospital. Inclusion criteria were: Length of ICU stay > 120 h (5 days), Sequential Organ Failure Assessment (SOFA) score [32] during ICU stay > 5, age ≥ 18 years and estimated life expectancy > 6 months. At the end of the ICU stay patients were approached if they were responsive (i.e. no sedation, fully conscious, no medical interventions). Patients were excluded if they were less than 18 years old, gave no written informed consent (unable or unwilling), were not expected to survive more than 6 months after hospital discharge, had an ICU stay equal or shorter than 120 h, had a SOFA score of 5 or less, were unable to complete questionnaires or had insufficient German language skills. The study physician explained the trial and handed out the study information brochure. Informed consent was obtained during the ICU stay from participants or their legal representatives. Participants were randomised to intervention or control group. They did not receive any incentives. The scientific study team prepared computer-generated permuted block randomisation with blocks of size 10 to ensure balance between groups over short time spans [33]. The responsible study physician received an opaque, sequentially numbered envelope supplied by the study team. The study physician opened the envelope after the participant's consent to take part in the study and explained the result of the randomisation. Blinding of the study personnel or the participants after randomisation was not possible due to the nature of our study. Nevertheless, the trust centre masked group allocation for the outcomes assessor during analysis of the primary outcome.

### Intervention

The intervention was a complex intervention including three components mentioned above. An intervention manual was compiled that specifies all procedures and contained the necessary instructions for action.

Shortly before ICU discharge, a first consultation took place. Participants were informed about the next steps: transfer to a regular ward, appointment to visit the follow-up clinic, telephone availability and monitoring by the study team, distribution of information material, e.g. ICU Steps brochure [34].

The ICU follow-up clinic visit was scheduled three months after ICU discharge. Three weeks before the visit the study nurse called the participants and reminded them of the upcoming appointment. The participants’ relatives were also invited to the appointment.

First, a brief assessment of the health condition and a screening of mobility was performed by the study nurse before the physician's consultation, including a blood pressure, oxygen saturation and heart rate measurement, and short current medical history. A standardised checklist based on questionnaires (Mini-Cog [35], PHQ-8 [36], GAD-7 [37], PTSS-10 [38], structured questions on symptoms of dysphagia and neuropathy [39]), were completed. Participants were motivated to self-complete the screening instruments and only received support by the study nurses, if they needed it (which was often the case). After completion, the study nurses evaluated the screening questionnaires. In case of strong deviations, irregularities in the preparation and peculiarities concerning the patient, they made memos in preparation for the consultation with the physician. The results of the questionnaires together with the memos then formed the indications for further questions from the physician. Thus, possible actions (e.g. further diagnostics, revision of medication, referrals) could be initiated immediately. During the consultation, participants and – if present – their next of kin were able to talk about the experiences at ICU and to ask questions. In the further medical examination, all areas (cognition, psyche, body, social, well-being) were examined. All participants were offered a visit to the ICU facilities and participation in a self-help group. The study physician wrote a physician's letter summarising the most important information about patient's health condition. In addition, the letter contained recommendations on further therapy measures, a description of the referrals issued and notes for the general practitioner. Information on e.g. social security issues, sleep hygiene or stockings were given to almost all participants. This letter was discussed intensively with the participant and if present next of kin and there was again room for questions. Referrals to therapists or specialists were organised.

At the latest four weeks after the ICU follow-up clinic visit, the study physician called the participants. During the telephone call, the state of health and, if applicable, the progress of the referrals were queried. If new impairments were detected, a referral to a GP visit was made or an additional ICU follow-up clinic visit was offered (case-by-case decision). The telephone monitoring also served as a monitoring tool for the implementation of the recommendations made. Furthermore, participants and relatives had the opportunity to call the ICU follow-up clinic during office hours to speak with the staff.

### Control treatment

Participants enrolled in the control group received usual care without any additional information or consultation or monitoring until the follow-up assessment six months after discharge. Usual care in this context means that patients receive rehabilitation during and after a longer stay in hospital. The aim of rehabilitation in Germany is to strengthen physical, mental, social and vocational skills as well as self-determination and equal participation in all areas of life [40]. Rehabilitation in Germany is subdivided in different phases [41]. Early rehabilitation takes place in phases B and C in particular (phase A comprises the acute treatment). In phase B, patients usually still require intensive medical treatment, e.g. need to be ventilated. In phase C, they can already cooperate in therapy, but must continue to receive medical care and nursing [41]. Early rehabilitation is part of (usual) hospital treatment and is covered by health insurance if medically necessary [42]. It is started during acute inpatient treatment and can be continued in specialized facilities. The goals are, in particular, early mobilization, avoidance of later complications, and clarification and planning of further rehabilitation and care measures. A multidisciplinary team of physiotherapists, occupational therapists, speech therapists, neuropsychologists, social workers, specialized nursing and physicians work in these early rehabilitation facilities [43]. Depending on the severity of the permanent restrictions, those affected can then be reintegrated into working life, e.g., through follow-up rehabilitation and occupational rehabilitation measures, or receive benefits from long-term care insurance (e.g., long-term care allowance, day and night care) if the need for long-term care persists [24]. However, these efforts are not targeted to patients after ICU stay.

### Feasibility outcomes

A quantitative process evaluation was conducted using a logic model [28] to frame our evaluation questions [30]. The overarching questions were:

- Is a pragmatic RCT on the effects of the participatory developed ICU follow-up clinic feasible in terms of recruitment, randomisation, intervention delivery and follow-up?
- Is it feasible to care for patients in terms of improving physical functioning and mental health in a participatory developed ICU follow-up clinic?

To answer those questions, the following key domains of feasibility were assessed:

- Acceptability
  - ◦ Number of study participants divided by number of possibly eligible patients (consent rate)
  - ◦ Proportion of study participants who accepted their random group allocation (acceptance of randomisation)
- Fidelity
  - ◦ Number of study participants to whom all components of the intervention were delivered divided by all included study participants
- Completeness
  - ◦ Number of study participants lost to follow-up divided by all included participants (attrition rate)
  - ◦ Mean completeness of baseline measurements (on instrument level)
  - ◦ Mean completeness of follow-up measurements (on instrument level)
- Practicality
  - ◦ Proportion of participants needing assistance with questionnaires.

We predefined those domains and respective outcomes according to literature [44, 45]. We decided against certain thresholds because there will not be a single threshold above which the RCT is not feasible anymore.

### Effectiveness outcomes (targeted results of the intervention)

#### Primary outcome

Primary effectiveness outcome was physical HRQOL at six months after informed consent/ICU discharge assessed by the physical component scale (PCS) of the Short Form-12 self-report questionnaire (SF-12) [46] with a four-week recall period. Items were scored according to published algorithms (German norm values; resulting in a standard score with mean = 50 and standard deviation = 10) [47]. Scores range between 0–100, with higher values indicating higher HRQOL.

#### Secondary outcomes

The secondary outcomes encompassed physical functioning, mental and social impairments as well as the extent of ambulatory and stationary health care use after ICU discharge. In addition, HRQOL of next of kin was assessed using the SF-12 questionnaire (PCS and MCS). Primary and secondary outcomes are shown in Table 1.

**Table 1:** Overview of primary and secondary outcome assessment tools

**Primary Outcome (participants)**	**Measurement**	**Reference**
HRQOL, physical health	SF-12 (PCS)	[46, 47]
**Secondary Outcomes (participants)**		
HRQOL, mental health	SF-12 (MCS)	[46, 47]
Activities of Daily Living (ADL)	Barthel-Index (self-report and by proxy report)	[48, 49]
Physical functioning	Chair Rising Test	[50, 51]
Overall muscle strength	Hand grip strength assessment	[52, 53]
PTSD	Post-Traumatic Stress Syndrome 10-Questions Inventory (PTSS-10)	[38, 54, 55]
Symptoms of depression, panic attacks, psychosocial impairment	Short Form of the Patient Health Questionnaire (PHQ-9)	[37, 56, 57]
Health care use	13 items on frequency of visits to physicians from different specialties	
**Outcomes (next of kin)**		
HRQOL	SF-12 (MCS, PCS)	[46, 47]

*HRQOL* health-related quality of life, *PCS* physical component scale, *MCS* mental component scale, *PTSD* posttraumatic stress disorder

### Data collection and analysis

To keep the participant's strain to a minimum, we collected as much information as possible from the clinical information systems (CIS) and next of kin (e.g. contact information, disease- and therapy-related characteristics). At inclusion in the study, we recorded their baseline measurements (e.g. sociodemographic characteristics not available from CIS) and quality of life assessed in form of the EQ-5D Visual Analogue Scale (VAS) [58]. The study team considered EQ-5 D VAS as the most feasible measurement instrument to assess health-related quality of life due to its brevity to keep the burden on the participants as low as possible at this very moment.  At the follow-up assessment six months after discharge from ICU, we collected outcomes mentioned in Table 1. Trained study personnel handed out self-report questionnaires, provided standardized instructions and performed physical measurements. Missing data were minimized by having study personnel available at all times to check the completeness of the questionnaires or to answer participants´ questions. If participants were not able to visit the study centre, home visits were offered. If a home visit was not feasible, paper-based assessment with telephone support was considered. If a participant discontinued the trial before outcome assessment, only baseline data (e.g. sociodemographics, characteristics of the ICU stay and more details in Table 3) and the reasons for discontinuation were included in the analysis. All data were entered by the study team on site twice into a centralised database and checked for plausibility.

We compiled a statistical analysis plan (SAP) a priori that was already geared towards a large-scale study. Descriptive statistics were used to determine participant characteristics and to check their distribution at baseline in the intervention and control group. We calculated point and interval estimates where appropriate. Continuous data are presented as mean and standard deviation or median and interquartile range, depending on whether the values were normally distributed; normal distribution was tested with Shapiro–Wilk test. Categorical data are reported as counts and percentages. The primary outcome physical HRQOL was compared six months post randomisation between intervention and control group. As our overarching goal was to evaluate the feasibility of delivering an RCT comparing usual care with an ICU follow-up clinic, the study was not designed to measure differences between treatment groups (e.g. no power calculation). Therefore, no formal hypothesis testing was performed. Nevertheless, we undertook descriptive analyses on the outcome measures of interest. Data management was done with Microsoft© Access© 2016. Analyses were performed with IBM® SPSS© Statistics version 26.0 and 28.0.

### Deviations from the initial study protocol

We initially started our study in December 2019. Due to the COVID-19 pandemic and related restrictions imposed by the German authorities also on clinical research, we had to discontinue all our activities (e.g. recruitment, inclusion of participants, the follow-up clinic) on March 20, 2020. Consequently, we needed to restart the RCT, since the duration of the interruption was not foreseeable at this time. Intensive discussions among the study team and with the funding agency led to a reduction of planned participants to 40. We chose this number primarily for economic reasons, as limited duration of the project and limited budget did not allow a further extension to achieve a number of 100 participants. We were able to resume activities on June 15, 2020.

## Results

### Patient flow and feasibility outcomes

**Fig. 2:**
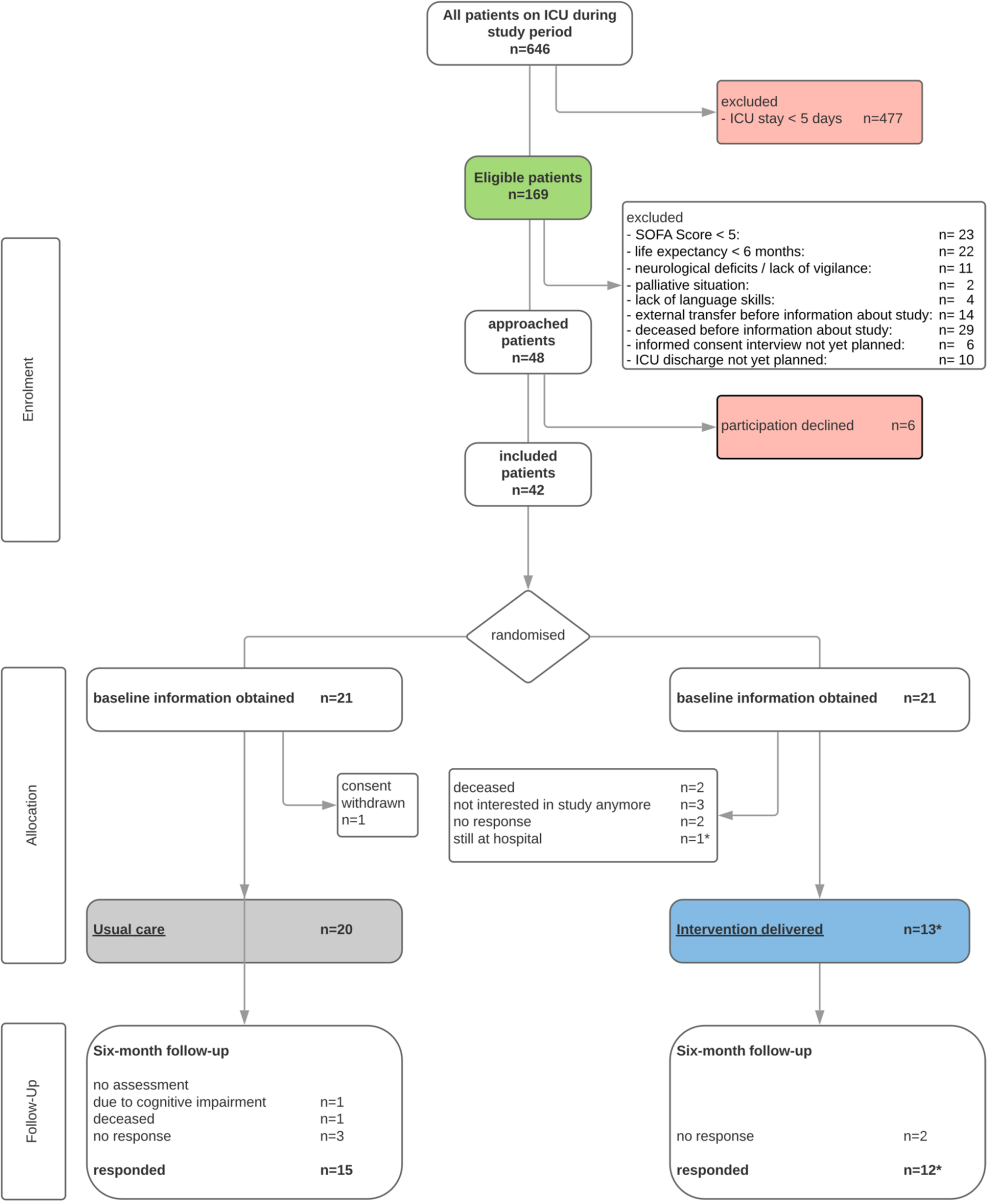
Patient-flow diagram. *one participant's hospital stay took so long that the ICU follow-up clinic visit would have been after the scheduled six-month follow-up, so both appointments were held at the same time

Participants were recruited from three ICUs (two medical and one surgical) at Regensburg University Hospital, Germany from June until September 2020. Based on a daily screening (60–90 min workload per day) two study nurses identified 169 eligible patients. 121 were excluded according to our exclusion criteria. A number of 48 potential participants were approached and informed about the study, of which 41 (85.4%) consented to take part in the study. Six suitable patients or their next of kin declined study participation with the following reasons: feeling too sick to participate, no interest in the study, not able to understand the language (*n* = 2, respectively). One patient withdrew his consent to participate after randomisation and was excluded for further proceedings and data analysis. The informed consent interviews lasted 20 min on average. All participants were randomised. All participants accepted the randomisation. Details on enrolment, allocation and follow-up can be found in Fig. 2.

For our predefined feasibility domains acceptability, fidelity, completeness and practicality, quantitative feasibility outcomes were measured alongside the RCT. All feasibility domains and the results for quantitative feasibility outcomes are shown in Table 2.

**Table 2:** Feasibility domains and respective outcomes

**Feasibility domain**	**measured by**	**%**
**Acceptability**	Number of study participants divided by number of possibly eligible patients (consent rate)	85
	Proportion of study participants who accepted their random group allocation	100
**Fidelity**	Number of study participants to whom all components of the intervention were delivered divided by all included study participants in the intervention group	62
**Completeness**	Attrition rate	34
	Mean completeness of baseline measurement instruments	100
	Mean completeness of all outcome measurement instruments	77
**Practicality**	Proportion of participants needing assistance with questionnaires^1^	Baseline: 27 Follow-up: 85

^1^Baseline: EQ-5D VAS, follow-up: SF-12, Barthel index, PHQ-D, PTSS-10

We were able to collect baseline values for all participants. Considering HRQOL measured by the EQ-5D VAS at baseline, 26.8% participants (*N* = 41) needed assistance in writing their value on the case report form. Approximately eight weeks after ICU discharge, 71% participants in the intervention group (*N* = 21) were reached out by phone to confirm the appointment for the ICU follow-up clinic visit. Two participants died in the meantime, three cancelled the further study participation actively. Two participants could not be reached, one was still treated at a hospital. The ICU follow-up clinic visit took place 11–12 weeks after discharge from hospital (mean/median 83/75 days after randomisation). There were some postponements due to a longer stay in rehabilitation. One participant’s hospital stay took so long that the ICU follow-up clinic visit had to be combined with the six-month follow-up assessment. The ICU follow-up clinic visit took 45–90 min (mean/median: 60 min), of which 15–60 min (median 30 min) were attended by the physician. Most participants were assisted from study personnel for completing the questionnaires. After the short evaluation by the study nurse, the results were discussed and recommendations for action assessed: Referrals to specialists (e.g. neurology, diabetology, gastroscopy) were issued eight times, referrals to therapy (e.g. physical therapy, counselling, podiatry) 21 times. For all participants, drug therapy was reviewed. The self-help groups were offered twice and due to no participants, the third meeting was cancelled in advance. Reasons for non-utilisation were a too great distance from home, no need due to improved health status or the effects of the COVID-19 pandemic (travel restrictions, fear of infection in the hospital). None of the participants took advantage of the offer to visit the ICU facilities.

Approximately three weeks after the ICU follow-up clinic visit, the study physicians conducted a telephone monitoring to discuss the current state of health and further therapy. Due to the COVID-19 pandemic, additional network meetings and events could no longer take place after the initial networking event in September 2019. The newsletter for health care providers envisaged in the original concept was not implemented. Moreover, the conceived referral list (i.e. information on therapists or physicians specialized in after ICU treatment) was not feasible because the catchment area of the study participants was too large (participants’ distance from home to follow-up clinic 6–310 km, mean 79 km).

Out of 41 participants with data from baseline assessment, we were able to re-assess 27 participants (66%) at the six-month follow-up. Attrition rate was 8/21 (38%) in the intervention group and 6/21 (29%) in the control group. In total, three participants died in between the six months after ICU discharge, ten participants did not respond or had no interest in the study anymore. No participant in the intervention group died in between the intervention and six-month follow-up. More details can be seen in Fig. 2. Thirteen participants were followed-up in the ICU follow-up clinic. Most of them needed assistance for completing the follow-up questionnaires. Six participants were visited at home. If even home visits were not feasible due to health status of the participant or the distance to the hospital, paper-based assessments with telephone support were conducted and thus the physical tests (grip strength and chair rising) could not be performed. Detailed completeness of the outcome measurement instruments are shown in Table 2 of the supplement S1. In particular, physical assessments and data collection from next of kin resulted in decreased completeness. The pilot RCT was duly concluded on 31/05/2021.

### Participant characteristics

The study sample consisted of 15 women (37%) and 26 men (63%). Male participants were overrepresented in both the intervention (*n* = 12) and the control group (*n* = 14) at baseline. Two out of 13 participants without long-term nursing care grade^1^ upon baseline assessment had a long-term care grade at follow-up, four participants had a long-term care grade both at baseline and at follow-up. The majority of participants (64%) graduated from middle school. Only 17% percent had a higher education such as college or university degrees. Twenty-two participants (54%) were not employed prior to the ICU stay, fifteen (37%) were full-time employed. An imbalance existed between the intervention and control group in terms of employment (33% vs. 60%). Median duration of ICU stay was almost similar in the intervention and control group as well as the SOFA Score, Barthel-index, number of participants with mechanical ventilation and mean HRQOL score. Duration of mechanical ventilation was longer in the control group than in the intervention group (median 271 h compared to 184 h). Further, extracorporeal life support measures (ECMO, dialysis) were applied more often in the control group. Considering the baseline characteristics, there was an imbalance between the intervention and control group. Further imbalances in baseline characteristics were seen for gender and primary diagnosis. More details of baseline characteristics stratified for intervention and control group (usual care) are shown in Table 3.

**Table 3:** Baseline characteristics of study participants

**Characteristics**	**Usual care (*N* = 20)**	**Intervention (*N* = 21)**	**Both groups (*N* = 41)**
Male participants, *n* (%)	14 (70.0)	12 (57.1)	26 (63.4)
Mean age (SD)	58.1 (15.8)	58.5 (12.3)	58.3 (13.9)
min–max, years	23–83	35–84	23–84
**Education, n (%)**			
No high school degree	1 (5.0)	1 (4.8)	2 (4.9)
General secondary school	16 (80.0)	11 (52.4)	27 (65.8)
Secondary school (Realschule)	2 (10.0)	6 (28.6)	8 (19.5)
Grammar school (Gymnasium)	1 (5.0)	3 (14.2)	4 (9.8)
**Vocational qualification, *n* (%)**			
None	4 (20.0)	2 (9.5)	6 (14.6)
Vocational school (Berufssschule)	4 (20.0)	3 (14.3)	7 (17.1)
Apprenticeship (Lehre)	11 (55.0)	10 (47.7)	21 (51.2)
Technical college (Berufsfachschule)	-	2 (9.5)	2 (4.9)
University	1 (5.0)	4 (19.0)	5 (12.2)
**Employment before ICU stay, *n* (%)**			
None	8 (40.0)	14 (66.7)	22 (53.7)
Part-time	2 (10.0)	2 (9.5)	4 (9.8)
Full-time	10 (50.0)	5 (23.8)	15 (36.5)
**Inclusion criteria**			
Median ICU stay (IQR), days	12.5 (9.0–27.0)	13.0 (8.0–18.5)	13.0 (9.0–25.5)
Median SOFA (IQR), highest score value during ICU stay	8.5 (7.0–11.8)	9.0 (8.0–12.5)	9 (7.5–12.0)
Median Barthel-index at transfer from ICU to normal ward (IQR), N*	62.5 (12.5–81.25), 14	60 .0 (30.0–80.0), 18	60.0 (30.0–80.0), 32
**Primary diagnoses**^a^, *n* (%)			
Cardiovascular	9 (45.0)	8 (38.1)	17 (41.5)
Pulmonary/ respiratory	6 (30.0)	2 (9.5)	8 (19.5)
Gastrointestinal	4 (20.0)	1 (4.8)	5 (12.2)
Cancerous condition	1 (5.0)	2 (9.5)	3 (7.3)
Other^b^	-	8 (38.1)	8 (19.5)
**Aggregated diagnostic categories**, *n* (%)			
Surgical	8 (40.0)	9 (42.9)	17 (41.5)
Medical	12 (60.0)	11 (52.4)	23 (56.1)
Trauma	-	1 (4.8)	1 (2.4)
**Ventilated during ICU stay**, *n* (%)	17 (85)	18 (85.7)	35 (85.4)
Median duration of mechanical ventilation (IQR), hours	271 (121.0–551.5)	184 (103.5–225.75)	198.0 (109.0–403.0)
**Extracorporeal life therapy**^c^, *n* (%)	9 (45.0)	7 (33.3)	16 (39.0)
ECMO	3 (15.0)	4 (19.0)	12 (29.3)
Dialysis	7 (35.0)	5 (23.8)	7 (17.1)
**Mean EQ-5D-VAS at baseline, (SD), min–max, score value**	51.7 (25.7), 10–95	52.1 (22.9), 5–90	51.9 (24.0), 5–95

*SD* standard deviation, *IQR* interquartile range

^*^N differs from respective total Ns for intervention and usual care

^a^Primary diagnosis classified according to ICD-10 representing the major disease upon admission to ICU

^b^Other diagnoses included musculoskeletal diseases, (bacterial) infections, urogenital diseases and trauma

^c^More than one extracorporeal therapy was possible at the same time

### Effectiveness outcomes (targeted results of the intervention)

#### Primary outcome

Our primary outcome (physical health-related quality of life) was lower in the intervention (mean 34.3 ± 10.8, min. 20.6, max. 53.6) than in the control group (mean 39.3 ± 11.0, min. 26.3, max. 56.0).

#### Secondary outcomes

Participants in the intervention group had more favourable outcomes in HRQOL (SF-12, mental health, MCS), activities of daily living (Barthel index) or indication for depressive disorders than the control group. The mean values for hand grip strength as an indicator of overall muscular strength were 28 kg and did not differ between intervention and control group. There was almost no difference for mental symptom-related difficulties in daily and working life (short form PHQ-D). Physical functioning (chair rising test) was better in the control group than in the intervention group (16 vs. 24 s for getting up five times from a chair without support). HRQOL of the relatives of the former ICU patients was better in the intervention group than in the control group (54 vs. 49). More details on secondary outcomes are presented in Table 4.

**Table 4:** Secondary outcomes in the intervention and control group (usual care)

**Outcome measure**^1^	Usual care (*N* = 15)	*n*	Intervention (*N* = 12)	*n*
**SF-12, MCS score**	44.4 (12.4), 23.4–59.8	14	49.0 (11.1), 35.7–65.6	11
**Barthel index**^a^, **median (IQR)**	90.0 (70.0–100.0)	5	95.0 (66.3–100.0)	12
**Chair rising test, median (IQR), seconds**^b^	16.0 (9.5–22.5)	6	20 (12.0–32.5)	6
**Hand grip strength, kg**^c^	28.3 (13), 11–47	11	28.5 (15.7), 7–52	7
**PTSS-10 score**	27.4 (13.3)	15	25.8 (10.6)	12
Indication & strong indication for PTSD according PTSS-10, n (%)	9 (60.0)	15	6 (50.0)	12
**PHQ-D (short form)**				
Minimal to moderate^d^depressive condition, n (%)	12 (80.0)	15	10 (83.3)	12
Moderately severe to severe depressive condition, n (%)	3 (20.0)		2 (16.7)	
Strong and very strong mental symptom-related difficulty^e^in daily and working life, n (%)	4 (26.7)	15	3 (25.0)	12
Panic attack during last four weeks, n (%)	4 (26.7)	15	3 (25.0)	12
**HRQOL next of kin**				
SF-12, PCS score	48.7 (7.4), 37.5–57.0	10	53.1 (4.9), 46.5–58.0	4
SF-12, MCS score	38.5 (11.4), 12.5-50.3	10	47.3 (10.0), 34.0–57.8	4
**Health care use (any visit to the following specialties), n (%)**^f^		15		12
General medicine	14 (93.3)		10 (83.3)	
Internal medicine	9 (60.0)		7 (58.3)	
Otolaryngology	5 (33.3)		9 (75.0)	
Neurology	9 (60.0)		2 (16.6)	
Dental medicine	7 (46.7)		3 (25.0)	

^1^Mean (standard deviation) and minimum–maximum, if not stated otherwise

^a^Self-reported; cut off values for Barthel index: 0–30 total dependency on care, 35–80 moderate dependency on care, 85–95 slightly dependency on care, 100 independence [48, 49]

^b^A duration of more than 11 s is considered an indicator of increased risk of fragility fractures [60] or even a higher risk of mortality [61]

^c^Maximum value out of three measurements on each hand [62]

^d^Categories according to Kroenke and Spitzer [63]

^e^Categories according to Löwe et al. 2002 [37]

^f^Other utilization of medical specialties stated by the participants: dermatology, radiology, surgery, urology, ophthalmology, gynaecology, psychotherapy, orthopaedics; multiple answers were possible

## Discussion

To the best of our knowledge, this is the first study investigating the feasibility and potential effects of a complex intervention in form of an ICU follow-up clinic for ICU survivors in Germany.

### Feasibility

Principal findings of this study were a consent rate of 85%, a fidelity rate of 62% and an attrition rate of 34%. Within 12 weeks, we recruited and successfully randomised 42 participants for the pilot study, i.e. 21 per arm. The participants accepted randomisation. One participant revoked his consent to participate in the study after assignment to the control group; the designated procedures to remove participant’s data worked. Some components of the intervention (e.g. self-help groups) could not be carried out largely due to COVID-19, which resulted in the 62% fidelity rate. Loss to follow-up mainly resulted from the fact that the participants were not (any longer) reachable. We know of three deceased participants during our study period. Data from other studies on ICU aftercare indicate that e.g. one-year-mortality is between 16–77% [64]. Further, the expected mortality among critically ill patients should be considered in the sample size calculation for a large-scale trial. Regarding the inclusion criteria and target group for an ICU follow-up clinic, patient groups for which dedicated follow-up programmes already exist (e.g. after organ transplantation, after a stroke or with cancer treatment) might not be included in a large-scale trial assessing the effects of the ICU follow-up clinic. Baseline data collection was successful and complete. The imbalance of baseline characteristics of the study sample might be due to the small sample size. Completeness of assessment of the primary outcome among all participants with completed follow-up was very good (93%); however, data which were assessed through physical examinations or data collected from next of kin was not complete in about 45% and 48% of cases due to logistic reasons. It is interesting to note that the relatives of the study participants often did not answer the questions on health-related quality of life (SF-12 MCS, ca. 50% completeness). The respondents might have felt under pressure at this moment or did not want to give "wrong" statements (social desirability). This could be addressed by better informing the patients’ relatives about the study already in advance, by explaining why their health is also important.

We were able to deliver the intervention to 14/21 study participants in the ICU follow-up clinic (see Fig. 2). The ICU follow-up visit took 60–90 min. In the context of a large-scale study (or as part of routine care), adjustments would have to be made in time management. One suggestion would be that patients could fill in the questionnaires before the ICU clinic follow-up visit in order to make this time window (20— 30 min) more effective. Regarding the six-month follow-up assessment, one should consider that particularly participants from the control group might have no incentive to travel to the study centre, especially if they live at a distance. Home visits took up a lot of resources that may not be available in everyday care and should be reconsidered for the effectiveness study. Of course, this could reduce the number of participants assessed in-person in a large-scale trial.

At baseline, 27% study participants needed support to point their value on the EQ-5D-VAS at that time. Further, 85% of the participants needed help in filling out the follow-up questionnaires, which would mean high effort for a larger study. Those consisted of five measurement instruments (SF-12, PHQ, PTSS-10, Barthel index, health care use), which may explain the high amount of assistance needed. We did not carry out a detailed analysis of the feasibility or acceptance of the single measurement instruments used. However, in the interest of data economy, minimising the amount of missing values and reducing the burden on patients, we suggest that the selection and composition of the measurement instruments in the large-scale study should be chosen even more carefully. Choice of outcome measurement instruments for a future trial should consider recent developments, in particular regarding core outcome sets [65–67], and should also take into account that the MCS and PCS of the SF-12 may not be independent components and therefore need to be interpreted with caution [68].

### Signals for effectiveness of the intervention

With regard to the effectiveness of our intervention, the pilot trial with 41 participants showed lower physical HRQOL (primary outcome) in the intervention group compared to the control group (mean 34.3 vs. 39.3). However, the mental HRQOL of the participants in the intervention group (mean 49.0) was higher than that of the control group (mean 44.4). It can be hypothesised that the intervention had a greater impact on the participants’ mental health than on their physical health. This is in line with previous findings from the PRacTICaL study [22]. However, as mentioned above those component scale values need to be interpreted with caution. A recent systematic review synthesised subject-related outcomes of post-ICU follow-up clinics: Rosa et al. [16] indicated also that ICU follow-up clinics are associated with improved mental HRQOL outcomes. In our study, we also explored patients’ perceived benefits of the intervention using qualitative interviews. The results will be published elsewhere.

### Deviations from the initial study protocol and its implications

The initially planned sample size of 100 participants had to be reduced due to the impacts of the COVID-19 pandemic. However, the final sample size followed recommendations for pilot trials if the effect size is unknown but expected to be small [69]. The reduced sample size although might have led to the imbalance between intervention and control group, so outliers might influence outcome evaluation. Therefore, we could not assess whether the ICU stay or an underlying disease (as the reason for the treatment) of the respective participant caused the recorded impairments.

Second, participants did not attend the planned self-help groups, so we were not able to actively assess acceptability or feasibility of this intervention component. Perhaps not attending the groups reflects a form of non-acceptance of this component.

Third, a network of health care professionals could not be implemented due to the impact of the pandemic; cross-sector collaboration for follow-up care could not be assessed.

Fourth, our SAP was already geared towards a large-scale study. Therefore, parts of it could not be implemented e.g., regarding the data analysis no intention-to-treat approach could be followed to analyse the effect of the ICU follow-up clinic on HRQOL as primary outcome and the secondary outcomes due to the small sample size and the nature of the conducted feasibility study. In addition, a sensitivity analysis was not suitable with regard to this data basis.

### Strengths and limitations

Our complex intervention (ICU follow-up clinic) was developed in a participatory multi-stakeholder process. This enabled us to consider as many different perspectives as possible. The intervention was subsequently implemented in an RCT and the entire concept was tested for feasibility. The external evaluation as well as the (initially blinded) outcomes assessment further improved the quality of the here presented concept and study. This should be also considered in the large-scale trial in order to evaluate the effectiveness of the ICU follow-up clinic. In this study, we were not able to identify the exact healthcare trajectories of each participant after the ICU stay and we did not gather data on healthcare use before their critical illness. Therefore, no comparison on healthcare use before/after critical illness was possible. Even in light of COVID-19, we were able to restart and manage the study, however with a reduced number of participants. Statistical testing regarding the RCT’s primary outcome was not intended per our study protocol [28]. The low number of feasibility studies of ICU follow-up clinics is remarkable. We contribute to this body of research by providing a pilot study that went through all the stages of a proper RCT, including an extensive feasibility analysis of the procedures. We have collected and reported on the decisive parameters that are mentioned in the literature on pilot studies [44, 45, 70, 71]. It seems essential to define clear feasibility criteria, measure relevant outcomes and evaluate the respective pilot study before a definitive trial on complex health care intervention in order to avoid “ineffective” effectiveness trials.

### Comparison to other ICU follow-up services feasibility studies

Previous studies on ICU follow-up clinics have mainly been conducted as full RCTs. Teixeira et al. [72] compiled an overview on effectiveness and feasibility of 30 studies on ICU follow-up clinics and did not find statistically significant effects in patients or their next of kin. They concluded that only individualized aftercare might be the right model for post-ICU patients. Six of the included studies were RCTs. However, none of these studies was a feasibility trial. In a systematic review of Schofield-Robinson et al. [73] on the effectiveness of ICU follow‐up services, the authors included five studies (four RCTs, one non‐randomised study) and concluded that there was only low-certainty of evidence that ICU follow-up services may make little or no improvements regarding HRQOL or PTSD at 12 months after ICU discharge. The systematic review of Geense et al. [74] on non-pharmacologic interventions to prevent PICS included ten pilot studies and five studies on ICU follow-up services but without overlap between those two groups. The four included studies on follow-up services are the same as in the systematic review of Schofield-Robinson et al. [22, 75–77]. One reason for the limited possibility to show effects of an ICU follow-up clinic on e.g. HRQOL or psychosocial wellbeing might be that only little effort has been dedicated to evaluate the feasibility of the proposed intervention and to perform a pilot study in advance. We identified three pilot studies [78–80] on ICU follow-up clinics (two RCTs, one cohort study, details in Table 5).

**Table 5:** Characteristics of other ICU follow-up pilot studies compared to our study

Characteristics	Henderson et al. 2021	Samuel et al. 2015	Bloom et al. 2019	PINA/our study
Year	2019–2020	12/2011–10/2012	05–10/2017	2021
Country	UK	UK	US	DE
Study Design	single centre cohort (5 sub-cohorts)	single centre RCT plus extra "low risk" control	single centre RCT	single centre RCT
Target Group	adult post-operative cardiac surgical patients and their caregivers	parents of paediatric ICU patients with at least 12 h ICU stay	adults admitted to the study ICU for at least 48 h with a predicted risk of 30-day same-hospital readmission of at least 15%	adult ICU (stay > 5 days) patients and their next of kin
Sample Size	113	78 + 131 (low risk controls)	302	41
recruiting time	not reported	not reported	not reported, most probably 6 months	12 weeks
Setting	Centre for heart and lung services	University teaching hospital	University hospital	University hospital; surgical and medical ICU
Aim of study	To describe long-term outcomes of cardiac intensive care unit (CICU) patients and their primary caregivers; and to explore the feasibility of implementing a complex intervention, designed to support problems associated with PICS and PICS-F,	To identify parents at risk for PTSD due to their children’s ICU stay	To increase the number of ICU recovery program intervention components received by patients due to the follow-up clinic (feasibility); and to decrease the frequency of 30-day hospital readmission (efficacy)	To explore and evaluate feasibility of an ICU follow-up clinic
Intervention	InS:PIRE programme, McPeak et al. [81]	follow-up clinic appointment 2 months after ICU discharge	10 components from discharge to 30 days after	ICU follow-up clinic
Components	five-week multidisciplinary peer support rehabilitation programme	review of current health condition of the child, reflection on the emotional experience of parents, access to further support services	inpatient visits, brochure, medication review, phone calls, ICU f/up clinic visit, assessment, neuro evaluation, referrals	assessment, medication review, social support, peer group
Aim of intervention	To ameliorate the signs and symptoms associated with PICS and PICS-F	To reduce parents' levels of posttraumatic stress, anxiety, and depression	To increase of use of intervention components / decrease of 30-day readmission	To increase HRQOL, physical functioning and psychosocial wellbeing
Follow-up	12 weeks after discharge (initial visit) and 3 & 12 Months after initial visit	not reported, most probably 6 months	not reported	planned: 6 months; actual: mean 198 days ~ 7 months
Willingness to participate	24%	52%	not reported, could be 232/302	85%
Acceptance of randomisation	not applicable	not reported	not reported, most probably 100%	100%
Adherence to the intervention	Initial uptake rate: 14%; uptake rates improved to between 25 and 30% following some changes in procedures	37%	not reported, 2/10 components completed (median)	mean 62%
Loss to follow-up	4%	24%	13%	34%
Completeness of baseline measurement	100% for patients / unknown for caregivers	100%	not reported; patients lost to follow-up were excluded from baseline analysis	100%
Main measurement instruments	HADS, EQ-5D	PAS, IES-R, HADS	readmission rate, death rate, health care utilisation	SF-12, PHQ, PTSS-10
Completeness of outcome measurement	54%	75%	not reported, most probably 100%	77%
Signals for effectiveness	HRQOL increased from 70 to 78	only small effect sizes in favour of the intervention for anxiety scores	time to readmission decreased in intervention group	Scores for mental HRQOL, ADL and relatives' HRQOL were higher in the intervention group

Henderson et al. [79] performed a single-centre feasibility cohort trial on a five-week multidisciplinary peer support rehabilitation programme for ICU survivors (complex intervention) and measured its impact on HRQOL, anxiety, depression, pain and caregiver strain through surveys. The intervention [81] contained a de-brief of the ICU stay, physical assessment and dedicated physiotherapy, review of medication, advice on social care support and peer support groups. Inclusion criteria were a minimum duration of mechanical ventilation of 48 h and a prolonged ICU stay (no further details provided). 27 participants (67% male, median age 66 years) were followed-up 12 weeks, three and 12 months after ICU discharge. The intervention showed to be feasible and safe in the clinical environment with a participation rate of 24% and an attrition rate of 4%. The initial visit at the clinic was 12 weeks after discharge compared to 20 weeks in our study. In contrast to our study, HRQOL was measured at three points (baseline, after 3 and 12 months) with the EuroQol Visual Analogue Scale (EQ-VAS). The mean baseline value was 70 (52 in our study) and improved to 78.

The feasibility study on a paediatric ICU follow-up clinic from Samuel et al. [80] measured the risk for posttraumatic stress, anxiety, and depression of parents whose children stayed on ICU for at least 12 h and evaluated if a follow-up clinic appointment two months after discharge decreased this risk. Half of the approached parents (52%) participated in their study. Randomisation worked and they did not observe significant differences between intervention and control group. The authors report a similar completeness of outcome measurement instruments to our study (75% vs. 77%). Uniquely, the study group included a low-risk group of parents (*n* = 131) which was not involved into the randomised trial but assessed in lights of acceptability measures. E.g. they were asked if the lack of invitation to the follow-up clinic was upsetting, which they denied. Further, 56% parents in the control group would have liked to visit the follow-up clinic. Identified barriers to visit the follow-up clinic were e.g. difficulties arranging time off work, travel costs or distance from hospital. In lights of signals for effectiveness, no significant differences were found between the intervention group and the control group six months after discharge.

Bloom et al. [78] performed a single-centre pilot RCT (“Vanderbilt study”) comparing an ICU recovery program to usual care. The program consisted of ten components (see Table 5) and were applied in between ICU discharge and 30 days after. Main feasibility outcome was the number of intervention components received. Further, readmission rate, death rate and health care utilisation were measured. Regarding the outpatient intervention components (the latter could be equivalent to an ICU follow-up clinic), only 8.1% attended an ICU recovery clinic appointment.

## Conclusions

Our participatory designed ICU follow-up clinic and a pilot RCT assessing its effectiveness were feasible in terms of acceptability, fidelity, completeness and practicality. Regarding the primary outcome physical HRQOL, there were no signals for improvement six months after discharge from the ICU. We recommend planning and conducting a multicentre study to evaluate the effects of our proposed ICU follow-up clinic, subject to aforementioned adjustments.

### Supplementary Information

**Additional file 1.**

## Data Availability

Data can be requested from the principal investigator (CA) upon reasonable request.

## Abbreviations

h: hours
HRQOL: Health related quality of life
ICU: Intensive Care Unit
Kg: Kilogram
Km: Kilometre
min: minutes
PICS: Post-intensive care syndrome
PTSD: Posttraumatic stress disorder
QoL: Quality of life
s: seconds
SAPS: Simplified Acute Physiology Score
SD: Standard deviation
SOFA-Score: Sepsis-related organ failure score

## Footnotes

1 In the German health care system 5 grades of nursing care (until 2016 known as levels of care) are assigned to persons, who cannot manage their daily lives independently and are dependent on the help of relatives or professional staff. The guidelines are anchored in the Eleventh Book of the Social Code Book (SGB XI). Further information on long-term care in Germany can be obtained elsewhere  [59].
