# Supplementary material for: Piloting an ICU follow-up clinic to improve health-related quality of life in ICU survivors after a prolonged intensive care stay (PINA): feasibility of a pragmatic randomised controlled trial
Source: BMC Anesthesiol. 2023 Oct 14;23:344. doi: 10.1186/s12871-023-02255-1 (PMC10576359; doi:10.1186/s12871-023-02255-1)
Supplement: Supplementary file 1 — Additional file 1. [file 12871_2023_2255_MOESM1_ESM.docx]

# Piloting an ICU follow-up clinic to improve health-related quality of life in ICU survivors after a prolonged intensive care stay (PINA): Feasibility of a pragmatic randomised controlled trial

Drewitz et al., BMC Anesthesiol (2023)

## Supplement 1 – Characteristics of follow-up assessment

Table 1 gives a detailed overview of the delivery of the follow-up assessment.

Table 1 Characteristics of the follow-up assessment

|  | **Usual care (N=15)** | **Intervention (N=12)** | **All (N=27)** |
| --- | --- | --- | --- |
| **Way of assessment, n (column %):**  **Participants assessed at the clinic** | 8 (53.3) | 5 (35.7) | 13 (48.1) |
| **Participants visited at home** | 3 (20.0) | 3 (21.4) | 6 (22.2) |
| **accompanied by next-of-kin** | Not recorded | Not recorded | 13 (48.1) |
| **Assessment via telephone** | 1 (6.7) | 0 | 1 (3.7) |
| **Paper based questionnaire by postal service** | 3 (20.0) | 4 (33.3) | 7 (25.9) |
| **Mean/Median (SD) time between randomisation and follow-up assessment (days), min-max** | 193.9/186 (18.5) 167-234 | 204.4/211 (27.6) 161-238 | 198.0/197 (22.9) 161-238 |
| **Mean/median duration of follow-up assessment (clinic or home visit), (minutes), mix-max** | 60.4/55 30-90 | 58.8/53 25-90 | 59.8/55 25-90 |

In Table 2 we show the completeness of every single measurement instrument which was used during the six-month follow-up assessment.

Table 2 Completeness of outcome measurement instruments

| **Outcome** | **Instrument** | **Completeness** | **%** |
| --- | --- | --- | --- |
| **HRQOL (participants)** | SF-12 | 25/27 | 92.6 |
| **Activities of Daily Living** | Barthel index | 17/27 | 63.0 |
| **Physical functioning** | chair rising test | 12/27 | 44.4 |
| **Overall muscle strength** | hand grip strength | 18/27 | 66.7 |
| **Psychiatric disorders** | PTSS-10 | 27/27 | 100 |
| **Mental disorders** | Short form PHQ-D | 27/27 | 100 |
| **HRQOL (next of kin)** | SF-12 | 14/27 | 51.9 |
